# Next generation sequencing as a panacea for antibiotic susceptibility testing: yea or nay?

**DOI:** 10.3389/fpubh.2025.1650925

**Published:** 2025-08-18

**Authors:** Alex van Belkum

**Affiliations:** ^1^ShanX Medtech, Eindhoven, Netherlands; ^2^Independent Researcher, Rijnsburg, Netherlands

**Keywords:** antibiotic susceptibility testing, next generation sequencing, antimicrobial resistance, critical assessment, *In Vitro* Diagnostics Regulation

## Abstract

Practical next generation sequencing (NGS) technologies are entering the high-throughput diagnostic clinical microbiology laboratory. Bacterial whole genome sequences (WGS) can be used for detection and identification of species and their (relative) quantification. Genomic relatedness and epidemiological spread of strains of microorganisms can be traced, in parallel with detection of virulence genes as well as genes involved in antimicrobial resistance (AMR). The latter potentially facilitates genomic antimicrobial susceptibility testing (gAST). AMR mechanisms and the genes involved are diverse and require dedicated supporting databases in order to be accurately detected by microbial genomics. The present document assesses the current position of NGS and gAST assays in the clinical microbiology laboratory and discusses their role in establishing a clinically actionable antibiogram which defines the spectrum of antibiotics to which a given microbial strain is susceptible or resistant. Key question is whether or not gAST has added value as compared to current AST methodologies. Full diagnostic implementation of gAST in the routine medical microbiology laboratory is as yet impossible. The technical complexity of gAST still needs a significant decrease, gAST data management needs to be improved and simplified, the timeliness of the gAST assays requires improvement, and costs need to go down. The throughput of genomic testing for large-scale routine medical-microbiological testing needs to be enhanced. Its clinical value needs to be better defined and requirements for optimal market access and acceptance should be further developed. When forthcoming gAST has been shown to be compatible with insurance and reimbursement budgets as well as microbiological QA/QC assessment and has been through the European *In Vitro* Diagnostics Regulation (IVDR) accreditation and/or US FDA approval, only then a more significant future role for gAST can be carefully considered. We should avoid that bureaucracy impedes the development of sequence-based AMR assessment. To date, routine gAST cannot do without combining it with rapid phenotypic AST.

## Introduction

### The classical clinical microbiology context

Clinical microbiology combines the specific and sensitive detection of disease-invoking viruses, bacteria, yeasts, fungi and parasites but still is a reasonably conservative expertise where diagnostic testing has been dominated by culture-based technologies for many decades (1–4). Especially in the field of bacteriology, microbial cultivation technologies developed by Pasteur and Koch in the nineteenth century continue to be important diagnostic workhorses (5). Obviously, culture-based bacterial detection has evolved, albeit slowly, and now, for instance, includes elegant assays that allow for sensitive cultivation in liquid culture media of the minute numbers of bacteria from septic patient’s blood (6). The performance and diagnostic value of color-mediated bacterial species identification directly on semi-solid culture media is non-disputed (7). Basic microbial cultivation has been supplemented with a variety of (bio-)chemical, immunological, physical and molecular methods for enhancing the sensitivity and specificity of microbial identification and characterization [for reviews see, (8, 9)]. Matrix assisted laser desorption ionization time-of-flight mass spectrometry (MALDI ToF MS) for instance has completely revolutionized bacterial identification over the past two decades. For a broad and detailed assessment of most if not all viable microorganisms in environmental and clinical samples so-called culturomics approaches have been designed and validated (10–12). Clinical microbiology identifies pathogens and allows for the selection of the best therapeutic drugs on a per patient basis.

### Clinical antibiotic susceptibility testing

An important medical-microbiological diagnostic application is the assessment of antibiotic susceptibility of bacterial isolates cultured from clinical specimens. Antibiotic susceptibility testing (AST) identifies antibiotics that are active against bacterial strains and as such guides optimal and accurate treatment of infections. AST should be rapid to allow the implementation of timely and correct treatment, it defines patient outcome by driving toward cure and it support antimicrobial stewardship (13). AST should be performed in real-time with ease of specimen collection, it should be affordable, sensitive, specific, user-friendly, rapid and robust, equipment-free or experimentally simple with limited hands-on time. This is exemplified, for instance, by lateral flow tests that can be used for the detection of extended-spectrum beta-lactames (14). AST should be easily deliverable to end-users and data communication should be secure and undisputed. These are the REASSURED criteria as defined by the World Health Organisation (WHO) (15, 16). Rapid AST (RAST) should provide high-quality results within 8 h although the current consensus is moving toward 2 or even less hours overall assay time. Many potential RAST methods that use a variety of chemical, physical and (micro)biological methods have been presented over the past decades but at present none of them is fully aligned with the REASSURED criteria [see (17–21) for technological reviews].

### Nucleic acid amplification testing-based AST

Molecular AST, mostly based on nucleic acid amplification testing (NAAT), was embraced by the diagnostic community over the past 30 years (22, 23). These tests detect genes (or diagnostic parts of those) that are fundamental to an AST phenotype and generate indirect proof of microbial antibiotic susceptibility. NAAT has been supplemented with nucleic acid sequencing, a diagnostic technology that is now in its fourth technical generation. Initial sequencing was based on a purely chemical methodology developed by Maxam and Gilbert (24). Next came enzymatic, DNA replication-based methods developed by Sanger et al. (25). Current automated high-throughput third and fourth generation applications [next generation sequencing (NGS)] allow for the collection of huge amounts of sequence data facilitating full microbial (and even eukaryote) genome sequencing (26–29). NGS requires sophisticated instruments and can be directly applied in the clinical microbiology laboratory (26, 30). Whether or not NGS is suited for genomic AST (gAST) is the core topic of this current manuscript, but this requires an introduction into the practice of current medical-microbiological AST methods first.

## Brief review of current AST methodologies

### Phenotypic vs. genotypic AST

Phenotypic methods define the direct physiological effect of an antibiotic on the viability of bacterial cells. Phenotypic methods often measure (lack of) cell density and division as expressed by changes in the number of viable cells present over time in a controlled environment with or without antibiotics. Phenotypes are measured by quantifying transmission of light through a bacterial culture or by cellular activity (changes in morphology, movement, metabolism, presence or absence of certain proteins etc). Second, indirect genotypic methods detect molecular markers associated with antibiotic susceptibility or resistance. A large variety of such tests has been developed, essentially for all microbial species and/or resistance mechanisms known (18, 31). The test format is mostly PCR-based although several tests depending on isothermal amplification technologies are available as well (32, 33). It is clear that such approaches only allow for the detection of previously known resistance markers and are ignorant with respect to synergistic or antagonistic interactions between markers or additional genetic elements. In brief, phenotyping directly assesses the functional ability of a bacterial cell or population to resist static or cidal antibiotic effects. Genotypic testing identifies potential for resistance, not defining whether this directly and knowingly translates to a survival advantage. The most obvious need in the field of RAST is the development of tests that can be applied directly to a clinical specimen, that can be performed at the point of care (PoC) (e.g., in the general practitioners office), that are easily scalable and flexible and that allow rapid adaptation of the antibiotics (or concentration thereof) to be tested *in vitro*. Such tests are in development but, again, do not yet meet all of the REASSURED criteria although some show a strong promise especially for (direct) urine testing (34–36).

### AST technology

It is important to define the currently used laboratory methods for AST since these define the Gold Standard to which all new methods will be compared (37). Often used phenotypic methods include automated high-throughput technologies developed and marketed by dominant *In Vitro* Diagnostics (IVD) companies such as Beckman-Coulter (USA), ThermoFisher (USA), Becton-Dickinson (US) or bioMerieux (France). Flagship technologies such as WalkAway, Sensititre, Phoenix and VITEK2, respectively, facilitate growth-based AST. When automated systems are not available or required (e.g., due to low(er) diagnostic throughput in smaller hospitals), manual technologies such as macro-broth dilution, disk diffusion or antibiotic gradient tests may be performed [e.g., (38)]. Next to these classical methods, many innovative technologies have been assessed over the past decades with regard to their technology readiness level (TRL), clinical validation status and time-to-results (39). This has led to various user’s encyclopediae for technology developers and clinical microbiologists to help them better understand the phenotypic RAST technology landscape and its developmental pipeline (20, 40, 41). Various novel technologies presently allow the assessment of antimicrobial resistance (AMR) at the level of single cells. These technologies are inherently very sensitive and sometimes even cover the detection of antibiotic heteroresistance (40, 42–45). Finally, classical nucleic acid sequencing but also NGS can be coupled to NAAT or used as single, stand-alone diagnostic tools (46–49). Below more details on the applicability of NGS in AST will follow.

## NGS for AST

### NGS technology

NGS can be used in a single assay to detect bacteria and microbiomes, to quantify bacterial cells, to search for virulence genes, to define epidemiological relatedness among bacterial isolates and to provide information on antibiotic resistance genes (50). The workflow for this type of analysis consists of practical short-read or long-read sequencing provided by Illumina (San Diego, US) or Oxford Nanopore Technologies (ONT, Oxford, United Kingdom), respectively. Sequencing will be performed on DNA extracted either from pure bacterial strains or all bacterial cells present in a clinical specimen [also known as microbiome sequencing (96, 97)]. This is followed by read- or genome-based informatics facilitating the interrogation of the data for gAST markers (51–55). This approach allows for the definition of the so-called resistome in bacterial whole genome sequences (WGS) as well as in microbiome sequencing datasets. The resulting resistomes cover both cultivable and non-cultivable bacterial species. Currently, this has generated insights in the global distribution of resistance genes (56), an overview of the spread of multi-drug resistant (MDR) bacteria (57), dissection of genetic resistance transfer and exchange networks (58) and the assessment of the global evolutionary dynamics of AMR (59).

### Use of NGS data

First and foremost, experimental NGS data need to be reliable and reproducible. As recent as in 2020, multi-centered studies revealed that NGS data were insufficiently robust [e.g., (60)], a problem that has not been uniformly solved recently (61). Fortunately, most recent reports show that Nanopore sequencing may now have the intrinsic reproducibility needed for routine clinical microbiology application of NGS data (62). For optimal use and insight, raw NGS data need to be transformed into assembled genomes. This interpretation requires software suites, most of which based on De Bruijn graphs [e.g., (63, 64)]. The quality of WGS assembly tools such as SPAdes, Velvet, ABySS and SOAPdenovo as well as several metagenomic assemblers (IBDA-UD, MEGAHIT, MetaSPAdes and MetaVelvet) was recently reviewed (46). Next to the assemblers an important role is played by search tools that can detect and identify specific AMR genes in metagenomic and WGS datasets [e.g., (65)]. Several bioinformatic tools have been designed to directly analyze resistance genes in raw NGS datasets, without prior genome assembly (66–68). These tools are at the heart of gAST. It has to be noted that these latter read-based methods have become popular in diagnostic testing since these tend to be less time-intense than the assembly-based methods (69). Currently, there is no data management protocol that is broadly accepted by all medical microbiologists involved in gAST.

### gAST databases

The entire gAST approach depends on the content and intrinsic quality of reference microbiological and gAST databases. In such databases all resistance genes, variants thereof and their associated phenotypic antibiotic resistance effect need to be well represented and strictly quality controlled. Databases should be able to detect AMR genes and mechanisms but also individual (nucleotide-specific) mutations contributing to AMR. The current spectrum of databases includes ResFinder, CARD, ARDB, ARG-ANNOT, NCBI’s AMR FINDER PLUS, FARME, SARG, BLDB and quite some more [for a review and a detailed explanation of all abbreviations see (70, 71)]. Databases can be generic, covering many bacterial species and even more resistance mechanisms and variants. In addition, gene- or resistance mechanism-focused databases for single or a few bacterial species also have great diagnostic value [e.g., the *Mycobacterium tuberculosis* specific WHO Mtb Mutation Catalog V2 (ISBN: 9789240082410) as a pertinent example]. The precise correlation between a resistome and the resulting minimal inhibitory concentrations (MICs) for all antibiotics used in clinical care needs further study and calibration [e.g., (72) as developed for *M. tuberculosis*]. Again, there is not yet a consensus model accepted by most microbiologists for which there is a variety of reasons. In some cases academic databases or bio-informatic services have been discontinued, in others the spectrum of species or AMR mechanisms included is too narrow. Costs, technical expertise and availability and lack of immediate clinical need are among the culprits.

Of note, all of the above—NGS and gAST technology, interpretation of data, and database management—has resulted in a huge body of academic literature already and thus far the text here has not yet mentioned the tremendous success of using NGS for *M. tuberculosis* AMR prediction, which has become the first-line testing modality, and represents a cheaper, faster, and safer way of gAST for Mtb than the conventional culture-based AST (73, 74). It has to be stated as well that there are many unmet clinical needs for classical AST in slow-growing fastidious microbes including mycoplasmata and fungi. Conventional testing also is problematic for inducible resistance mechanisms including metallo-beta lactamases in *Aeromonas* spp. (75). The value of culture-based AST is limited in these domains and clearly gAST has a great role to play in this space. Instead of suggesting that gAST is not ready for use, it has to be emphasized that it is working great in certain niches, but still needs improvement in the general areas including routine high-throughput testing. Concluding, there still is a clear lack of regulation and quality approved, commercially supported routinely available tools for gAST in day-to-day practice.

## Critical assessment of gAST

### Practical issues with microbiological NGS

Major issues with NGS are its significant consumption of time, its technical complexity (requiring input by high-level technologists), its data intensity (sometimes complicating the recognition of valuable versus less valuable data) and, hence, its relatively high costs. Improvements in cost-effectiveness and rapidity are urgently needed. As the result of NGS, large amounts of experimental data are generated, over 99.9% of which is redundant to gAST. This puts strict demands on data quality assurance and control (QA/QC), assay repeatability, availability of internal positive and negative controls, data storage and overall data security and management. As with any other microbiological diagnostic assay, bacterial taxonomic controversies need to be dealt with as well, including species identification as well as AMR gene—and overall genome—nomenclature (76, 77). It needs to be realized that, in principle, NGS does not distinguish between living and dead microorganisms and neither does it discriminate (innocent) bacterial contamination or colonization from genuine infections, phenomena that co-depend heavily on clinical context (e.g., prior infections or treatment thereof). Also, gene presence is not a guarantee for its expression and it has to be realized that many extragenic elements may influence gene expression as well (repressors, promoter mutations, multiplicity of plasmids etc) (78). Finally, there is a continuous need for supplementing both phenotypic and genotypic experimental data mining with bioresources (including, e.g., reference strains, clinical specimens, (artificial) microbiome compositions and enzymes) that can be used for QA/QC during microbiological NGS.

### Data and database management

gAST database development still suffers from the lack of representative and high-quality reference sequences including well annotated sequences for resistance genes and their many variants. Database development will remain an ever expanding activity that will never be finished and identification and characterization of new genes and variants will require continuous intellectual and capital investment. It is still difficult to distill resistance gene abundance from raw datasets, especially in metagenomic data where inter-species homologies for certain genes may be problematic. Furthermore, genotypic data will always need to be supported by phenotypic reference data. Given the wide array of methods, full concordance between phenotypic and genotypic approaches is presently incomplete at best. The European Committee on Antimicrobial Susceptibility Testing (EUCAST) and its USA-based counterpart the Clinical & Laboratory Standards Institute (CLSI), the two organizations governing clinical AST cut off values, have thus far failed to come up with consensus approaches for meeting this limitation (98). The EUCAST authors concluded in their position paper published in 2017: “*For most bacterial species there is currently insufficient evidence to support the use of WGS-inferred AST to guide clinical decision making. WGS-AST should be a funding priority if it is to become a rival to phenotypic AST.*” Little changed over the past 10 years. To date, in the mid-twenties of the 21st century, curation of databases is still problematic and quite a few of the databases are still biased toward human pathogens and their specific resistance mechanisms. It will require well-managed international collaboration and standardization to improve the curation pipelines and agreements. Moreover, longevity of databases seems to be strongly dependent on the continuation of academic grants or projects. When finances run dry, databases are either put on hold or terminated effectively. This is an unfavorable situation that can only be solved when (semi-) commercial parties become involved.

## Future challenges

### Improving the gAST system architecture

Clinical microbiologists involved in high throughput routine diagnostics prefer sample in – result out approaches. Hence, the final workflow for gAST requires the inclusion of direct sampling from clinical specimens or even patients. This would then probably result in a metagenomic NGS strategy in which essentially all nucleic acids in a sample will be sequenced. Pre-analytical methods for the suppression of unnecessary host genome sequencing are needed to make the sequencing more targeted and productive. Hybrid-based target capture and generic suppression of human DNA have been successfully applied (79, 80), but it may also be argued that including some host DNA in the NGS can also provide important information on host’ disease susceptibility (97). gAST at the PoC would be advantageous as well but this would require REASSURED-compliant portable equipment with a small footprint (81). Methods for accurate clinical interpretation of the data as well as tools for safe and secure data communication, either or not based on artificial intelligence, need further development and promotion. Machine learning (ML) for the prediction of phenotypes directly from genotypic data has been developed and this methodology will be important for future data interpretation (49). A problem with ML is its dependency on training data or existing databases. In the end, commercial versions of cartridges, laboratory instruments and additional hardware as well as the software packages need to be put through formal research and development trajectories in compliance with regulatory requirements (see below). Ultimately, the entire gAST workflow must be automated. This will require future attention to aspects as diverse as documentation, training and instruction, the use of internal and external process controls, seamless sample transfer, maintenance, backup to downtime of the equipments and cleaning protocols.

### Compliance with the European *In Vitro* Diagnostic Regulation

The EU *In Vitro* Diagnostic Regulation (IVDR, Regulation 2017:746) is applicable to essentially all *in vitro* diagnostic medical devices. IVDR establishes a risk-based classification system for such devices (Class A: low patient and public health risk; B: moderate patient and/or low public health risk; C: high patient and/or moderate public health risk; D: high patient and high public health risk). The IVDR mandates oversight, detailed technical documentation, and post-market surveillance based on a comprehensive quality management system (QMS). The QMS is not only critical for compliance, it also demonstrates regulatory readiness of device manufacturers. There is no obligation to use the European harmonized standard ISO13485:2016 QMS, but this standard does serve as a point of reference for organizations involved in the design, manufacturing, installation, and servicing of *in vitro* diagnostic devices. This internationally recognized certification is important as it aligns with patient safety, increased customer satisfaction, and regulatory compliance. IVDR assessment requires a Notified Body (NB) which is an organization designated by an EU Member State to assess the conformity of new with existing IVD products before the new one can be placed on the market (82). Regulatory agencies such as the US Food and Drug Administration (FDA) are updating their regulations to be consistent with the IVDR and other regulations in other parts of the world. Any gAST application to be brought into clinical practice will have to “survive” NB assessments and IVDR and FDA demands. It has been suggested that the new IVDR is detrimental to clinical microbiologists since it may limit the use of home-brewn, laboratory-developed tests that are important in the diagnosis of emerging or rare pathogens. In this way also the financial balance of microbiology laboratories might be affected (83). Others are more positive and provide guidelines for meeting the IVDR, even in case of laboratory developed tests (84, 85). Currently, there are no FDA or IVDR compliant gAST tests available. This will probably change in the not too far away future.

### Market access and market acceptance

gAST market access and market acceptance will probably hitchhike with other genomic microbiology applications but are first and foremost facilitated by the supposedly high quality of the new tests. This should be visualized by comparisons of the new tests with the reigning Gold Standard processes [e.g., (86–88)]. Market acceptance is then dependent on early adopting clients that confirm the published advantages of the new test and as such promote uptake by the entire diagnostic community. Obviously the safety and effectiveness of the new IVD devices shoud be warranted and test should be affordable and largely falling within the REASSURED criteria. Again, at present none of the gAST tests are actually close to the stage where they approach commercial market access despite a significant body of academic literature supporting their usefulness. The development of appropriate target product profiles for new gAST tests could help improve their design and applicability and with that customer acceptance (88, 89).

### Clinical impact

US Clinical Laboratory Improvement Amendment (CLIA) regulations establish quality standards for laboratory testing performed on human specimens (blood, body fluid, tissue etc). This is done for diagnosis, prevention, or treatment of disease, or assessment of health. CLIA requirements focus on laboratory processes and personnel, while IVDR compliance requires a new test to clearly show added clinical value. A CLIA Waiver is a certification that allows certain diagnostic tests to be performed in non-laboratory settings (e.g., general practitioner’s offices or pharmacies). These tests are simple and carry a small risk of producing erroneous results. Under these conditions, laboratories can legally examine persons through waived tests in order to assess health and treatment. The CLIA positioning needs to be defined by comparison and reproducibility studies. The intended use of a test should already be indicative of its prospective clinical value. Scientific and clinical validity need to be defined and have to be demonstrated in practice. This should be based on a review of the published data during routine diagnostic testing and a performance concept of equivalence and similarity (90). Clinical studies need to be of sufficient size and geographic and institutional diversity. Clinical impact studies for gAST are essentially lacking at this stage although in case of the detection of antibiotic resistant tuberculosis important steps have been taken (91). More studies where gAST is compared with reigning AST technologies in large-throughput routine clinical microbiology laboratories for positive gAST health-economic effects are needed.

## Conclusion

As mentioned above, gAST has been shown to be functioning very well in a variety of diagnostic niches. Still, both IVDR companies and (high-throughput) clinical microbiology laboratories are not ready for routine application of gAST yet. This is most obvious in resource limited settings but even in developed economies gAST may be hard to afford. The costs for gAST are not yet competitive with those of more classical AST formats (92–94). Further, the need for NGS laboratory expertise and equipment, management of rapidity, a lesser test complexity and improved data interpretation are not yet fool-proof and continuous system development is warranted. However, when costs go down and automation up, future integration of gAST in microbiological diagnostics and public health management is foreseen (95). Even the position of classical microbiological cultivation may then be disputed since NGS allows for characterization of all nucleic acid molecules present in a clinical sample. Such microbiome and “infectome” targeting strategies may in the end provide a cost effective diagnostic panacea.

The recent transition to stricter IVDR has been a challenge to manufacturers of IVDs. Many have had to make changes to their products and their quality management systems (QMS) in order to comply with the new regulation. It is also important to note that gAST will remain a dynamic methodology where many more near-future changes in technology, data management and data interpretation are foreseen. Regulators need to come up with a strategy that would allow a fluent way of integrating such changes while avoiding lengthy (and costly) validation and verification processes. At the same time, NBs have become significantly more busy posing another limitation on rapid market access for new AST methods. Finally, we do need to find an NGS method that allows for accurate MIC-level quantitative AST. Rapid reporting of results is of utmost important for reaching adequate clinical impact. Results should also be actionable and devoid of unnecessary jargon. Obviously, we do need to realize that classical bacteriology will never become redundant since we will always need viable bacterial strains for storage, historical comparisons and the development of biobanks that have a broad impact on all aspects on infectious disease management. Most importantly, the current global need for improved RAST should drive development toward excellent and cheap rather than all-encompassing and expensive. To date, gAST cannot replace phenotypic RAST.

## Data Availability

The original contributions presented in the study are included in the article/supplementary material, further inquiries can be directed to the corresponding author.
